# Linkages between environmental factors (WASH and energy) and Infant and Young Child Feeding practices in rural India: implications for cross-sectoral interventions for child health

**DOI:** 10.2166/washdev.2021.005

**Published:** 2021-08-16

**Authors:** Priti Parikh, Corina S. Kwami, Rajesh Khanna, Marie Lall, Hanimi Reddy, Lorna Benton, Sanjay Sharma, Virendra K. Vijay, Logan Manikam, Monica Lakhanpaul

**Affiliations:** a Engineering for International Development Centre, Bartlett School of Sustainable Construction, University College London, London WC1E 7HB, UK; b Civil, Environment and Geomatic Engineering, University College London, London WC1E6BT, UK; c Save the Children, Gurugram, India; d Institute of Education, University College London, London, UK; e International Center for Research on Women, New Delhi, India; f UCL Great Ormond Street Institute of Child Health, University College London, London, UK; g Indian Institute of Technology, Delhi, India; h UCL Institute of Epidemiology, 1-19 Torrington Place, London WC1E 7HB, UK; i Healthcare and Aceso Global Health Consultants Ltd, 3 Abbey Terrace, London SE2 9EY, UK; j Whittington NHS Trust, London, UK

**Keywords:** child health, energy, environment, feeding practices, India, WASH

## Abstract

As factors influencing the health and well-being of children are complex and cross-sectoral, integrated interventions are required to improve child health and hence address the Sustainable Development Goals. This paper explores linkages between environmental factors, feeding practices and potential infection pathways in India. The PANChSHEEEL project is a participatory interdisciplinary study, designed to explore HEEE (Health, Education, Engineering and Environment) factors influencing Infant and Young Child Feeding practices. This study uses data from observational transect walks and 445 household interviews from nine villages in Banswara district in India. Using the socio-ecological model, this study demonstrates how the lack of access to and quality of water resources, poor access to sanitation and hygiene practices, access to cooking fuels and flooding can influence feeding practices. The study finds that access to water, sanitation and cooking fuels can affect the preparation of food, contaminate water and food and place added time burden on caregivers. For infants, insufficient and contaminated water can lead to a higher risk of infection, diarrhoea and ultimately malnutrition. Recommendations include efforts to address waterlogging, promote safe storage of water, establish a water quality regime with stakeholders and develop appropriate, affordable and acceptable sanitation solutions.

## Graphical abstract

**Graphical Abstract:**
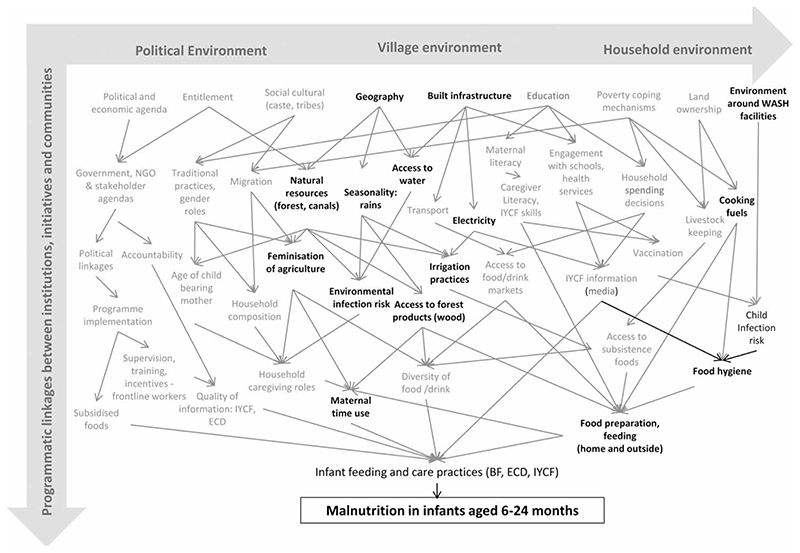

## Introduction

The United Nations Children’s Fund (UNICEF) Strategic Plan (2018–2021) advocates putting the needs of children at the heart of the United Nations 2030 Agenda to support the Sustainable Development Goals (SDGs) (UNICEF 2018). Parikh *et al*. (2021) demonstrated that action in WASH can benefit all 17 SDGs and 130 out of 169 SDG Targets. This demonstrates the wide-ranging benefit of improving WASH. Similar outcomes have been noted for improvement in energy systems with action in energy synergistic with 143 out of 169 SDG Targets (Nerini *et al*. 2018).

### Child health and feeding practices

The global burden of deaths is most acute for infants and young children in the first 1,000 days of life. Globally 149 million children under 5 were reported to be mildly, moderately or severely stunted (UNICEF World Health Organization (WHO) & World Bank 2019). Stunting and malnutrition are major health outcomes influenced by feeding practices. The causes are ‘multi-factorial and inter-linked’ spanning biological, social and environmental spheres (Cumming & Cairncross 2016).

The caregiver (mostly mothers) plays a strong role in feeding practices which are vital for achieving well-being, nutritional and development outcomes (Ngure *et al*. 2014). Feeding practices refer to the quality and quantity of food provided, preparation, storage and delivery. The physical environment together with the role of the primary caregiver has to be considered, as they influence child cognitive, sensorimotor, socioemotional development and growth (Ngure *et al*. 2014).

### Links between feeding practices, environmental factors and child health

At the national level, there is evidence of adverse health outcomes linked to environmental factors. The Lancet Child Development Series study of households in Bangladesh identified inadequate cognitive stimulation, stunting iodine deficiency and anaemia as adverse health outcomes associated with children’s play and feeding environments affecting 66% of households in Bangladesh (Lin *et al*. 2018). In the Philippines, data from 2,355 urban infants during the first 6 months of life found that mixed-fed and weaned infants consumed greater quantities of water and breastmilk supplements in the first 6 months of life and in so doing lose the protective effect of breastfeeding. This was common in highly contaminated settings and environments that lacked an in-house water connection or private excreta facility (Vanderslice *et al*. 1994). For babies that are on solid foods, with limited access to safe, potable water, the odds of contamination are higher (25 times) when utensils are not cleaned (Marino 2007).

Understanding and evidencing the linkages between Infant and Young Child Feeding (IYCF), environmental factors (e.g. contaminated water through ingestion, inadequate handwashing and cleaning) and child health can inform efforts to support caregivers’ capacity to provide appropriate feeding (WHO 2004). If the wider environment where children live exposes them to contamination, children will be at a higher risk of infections. The lack of availability of clean water may be due to adverse weather patterns fuelled by climate change and distance to water points. A systematic review by Brown *et al*. (2013) showed an increase in *per capita* water consumption when a water source was in close proximity to households with a knock-on impact on household environments, hygiene practices and reduced time burden for water collection – all factors affecting the well-being of children under 2 years. Water treatment through boiling of water has a high time and cost burden especially for women.

According to Cumming & Cairncross (2016), different WASH factors feature at various degrees of proximity to the outcome of stunting. Multiple aspects of WASH have been linked to four key areas of food and nutrition security: food ‘availability’, through water as a resource for agricultural production; food ‘access’, through household income diverted from food by the cost of obtaining water and ensuring adequate sanitation; food ‘stability’, through the economic shock of treating related infectious disease or associated inability to work and lastly food ‘utilization’, through the effect of WASH-related enteric infections on the body’s ability to utilise the available nutrients (Cumming *et al*. in press).

Lack of adequate toilets and lack of targeted child faeces management have been linked to higher incidence of diarrhoea. There is a need for further investigation of how the lack of (or use of) adequate toilets and how limited access to WASH facilities and handwashing activities (Morita *et al*. 2016) impact feeding practices and behaviours (Humphrey 2009).

The SHINE study examined environmental enteric dysfunction across the developing world and demonstrates that stunting may not be attributed to insufficient diet or diarrhoea alone but is embedded in a complex system of several environmental factors (Budge *et al*. 2019). The results did not support a direct link between feeding practices and water and sanitation, demonstrating the need for approaches which account for broader environmental factors (Budge *et al*. 2019). The WASH benefits trial also provides meaningful context surrounding why stunting is only modestly responsive to dietary interventions building on the literature that shows links between water quality, sanitation, and handwashing and child growth. Three randomised efficacy trials tested improved household-level WASH with and without improved IYCF on stunting and diarrhoea in Bangladesh, Kenya and Zimbabwe, demonstrating that baseline sanitation was a strong risk factor for stunting (Pickering *et al*. 2019). The literature review that grounded this work showed nearly all published studies have reported an effect of home-based water treatment and handwashing promotion on diarrhoea.

There is growing awareness of the effect of cultural and behavioural practices regarding childrearing but a need for further exploration of broader environmental factors that may influence behavioural practices (WHO 2004). For instance, the use of cooking fuels for food preparation is influenced by socio-cultural factors which in turn can influence feeding practices (Bartlett 2003). As the physical environment is shaped by complex infrastructural, cultural and social contexts, there is a need for interdisciplinary, community-based collaborative approaches that draw on a diversity of expertise in health, engineering and environmental studies to understand the linkages to feeding practices and child health (Kwami *et al*. 2019). There is a need for interdisciplinary interventions, which integrate environmental factors such as WASH to improve well-being for infants and children (Waller *et al*. 2020).

### Opportunities for an integrated approach to improving IYCF: India

The challenges associated with environmental factors and IYCF are particularly acute in South Asia, which has a high burden of suboptimal children’s diets, poor nutrition of women, lack of access to safe, potable water and lack of hygienic and sanitation practices in households and communities.

Chambers identified that despite many efforts to provide nutritious food to children, stunting in India has been persistent with WASH being ‘undernutrition’s blind spot’ (Chambers & Medeazza 2014). Lee *et al*. (2021) analysed Demographic and Health Survey data from India to examine associations between environmental factors and stunting. The study found that improved water and sanitation was associated with decreased odds of childhood stunting.

India launched an integrated policy for tackling malnutrition titled the Prime Minister’s Overarching Scheme for Holistic Nourishment (POSHAN) mission. The POSHAN mission is a multi-ministerial mission to monitor, supervise and fix targets, and guide nutrition-related intervention (Dasgupta *et al*. 2020). POSHAN mission’s goals are to improve child health through efforts to prevent and reduce stunting, undernutrition, anaemia and reduce low birth weight by taking a life cycle approach. The mission calls for the convergence of resources, skills and knowledge. It outlines elements of engagement and contributions of a wide range of departments and harmonisation of policies such as National Health Mission (NHM), Clean India Mission, National Rural Drinking Water Program, Infrastructure Development Program and Health Services and Delivery Research. The implementation of the POSHAN mission is a national mandate, with state-level actions to support capacity at a local level. This requires regional contextually specific evidence to co-develop tailored interventions in partnership with local communities. This study is timely, as it supports the ambitions of the POSHAN mission by providing local evidence and insights required to develop multi-faceted interventions to ensure impact at grassroots.

The evidence base that this paper builds upon suggests reframing the challenges to achieving child health through the linkages between IYCF practices and environmental factors. These factors are shaped by the local environmental and social contexts and require a framework that can effectively articulate those multiple and complex links. This would support shift the focus of current interventions beyond adequate quantity and quality of food to holistic interventions that account for the environmental context (Chambers & Medeazza 2014). A holistic approach to research and programme design of interventions that consider environmental factors is vital to improve IYCF practices. The WHO 2016–2030 global strategy aspires for evidence-based interventions that rely on community engagement and multi-sectoral enablers to address social determinants of health (WHO 2015). An integrated socio-ecological approach towards Health, Education, Engineering and Environment (HEEE) determinants of IYCF practices is necessary for sustained impact (Bhutta *et al*. 2013).

The PANChSHEEEL (Participatory Approach for Nutrition in Children: Strengthening Health Education Engineering and Environment Linkages) project is an interdisciplinary study designed to explore HEEE factors influencing IYCF practices and nutrition in India. The study developed an approach that was socio-culturally appropriate, tailored, innovative and integrated package to support optimal IYCF practices for children in rural India aged 6–23 months in close partnership with local communities and development partners (GCRF 2019). Using observational data from transect walks combined with evidence from household interviews, this study deepens current knowledge and understanding of linkages between environmental factors and IYCF in rural communities in the state of Rajasthan in India.

## Methods

The PANChSHEEEL project included an interdisciplinary team from University College London (UCL), Save the Children India (SCI), Indian Institute of Technology, New Delhi (IIT-Delhi) and Jawaharlal Nehru University, New Delhi (JNU). Ethical clearance was obtained from both the University College London Ethics Committee in the United Kingdom and Sigma- IRB in India.

### Study design

**Figure 1:**
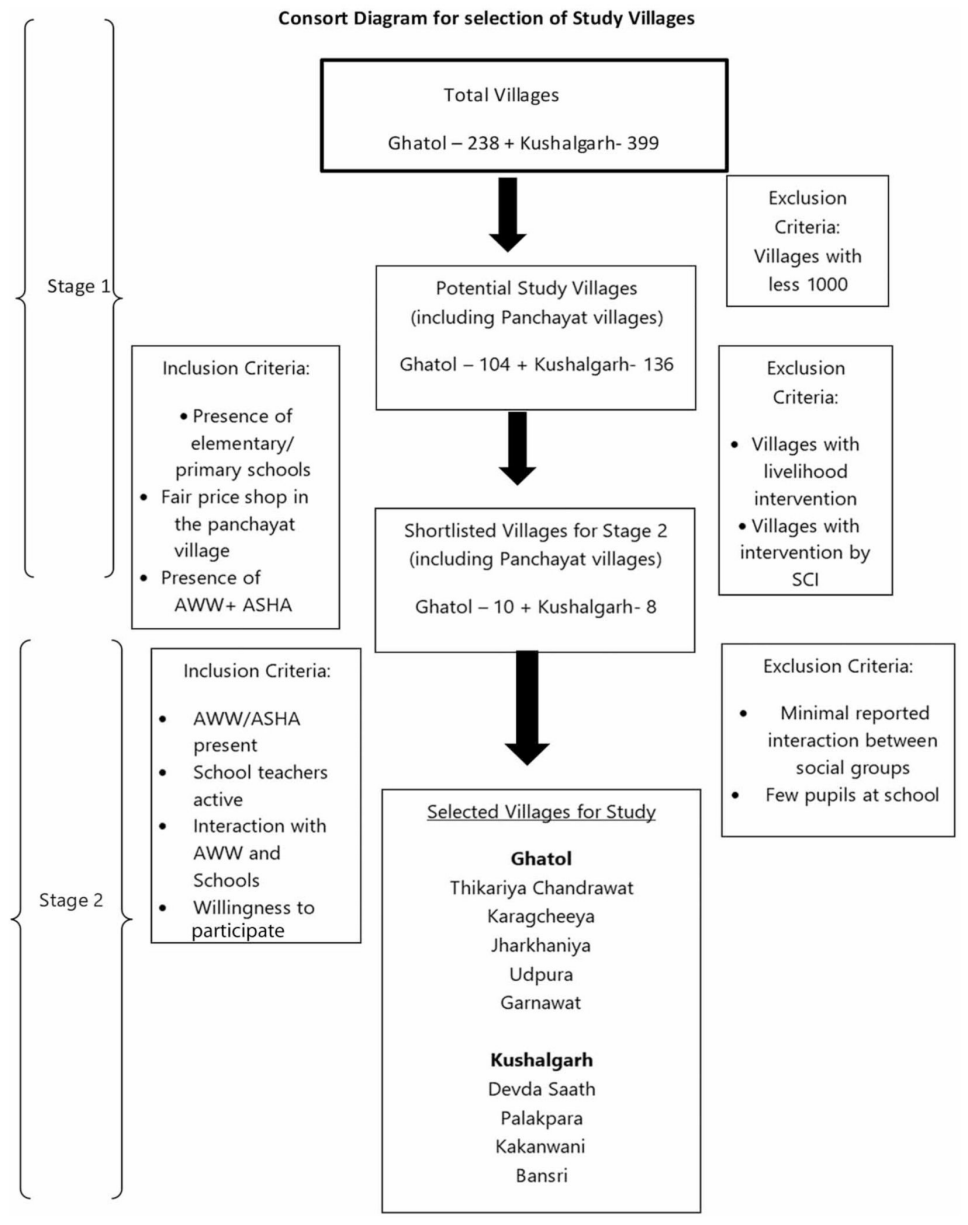
Consort diagram for selection of study villages (GCRF 2019).

This study was conducted in two blocks (Ghatol and Kushalgarh) in Banswara district, Rajasthan, India. This district is located on the Mahi River basin with two distinct divisions: canal and non-canal area. One block each was selected from the canal area (Ghatol) and non-canal area (Kushalgarh) to provide a diversity of water access scenarios and agricultural patterns. A consort diagram (Figure 1) shows the design and process for selecting the villages from these two blocks and the sampling frame. A total of nine villages were selected to include five from the Ghatol block and four from the Kushalgarh block. A full description of the village selection process, block selection and shortlisting is referenced in the wider study (GCRF 2019).

### Conceptual framework

**Figure 2:**
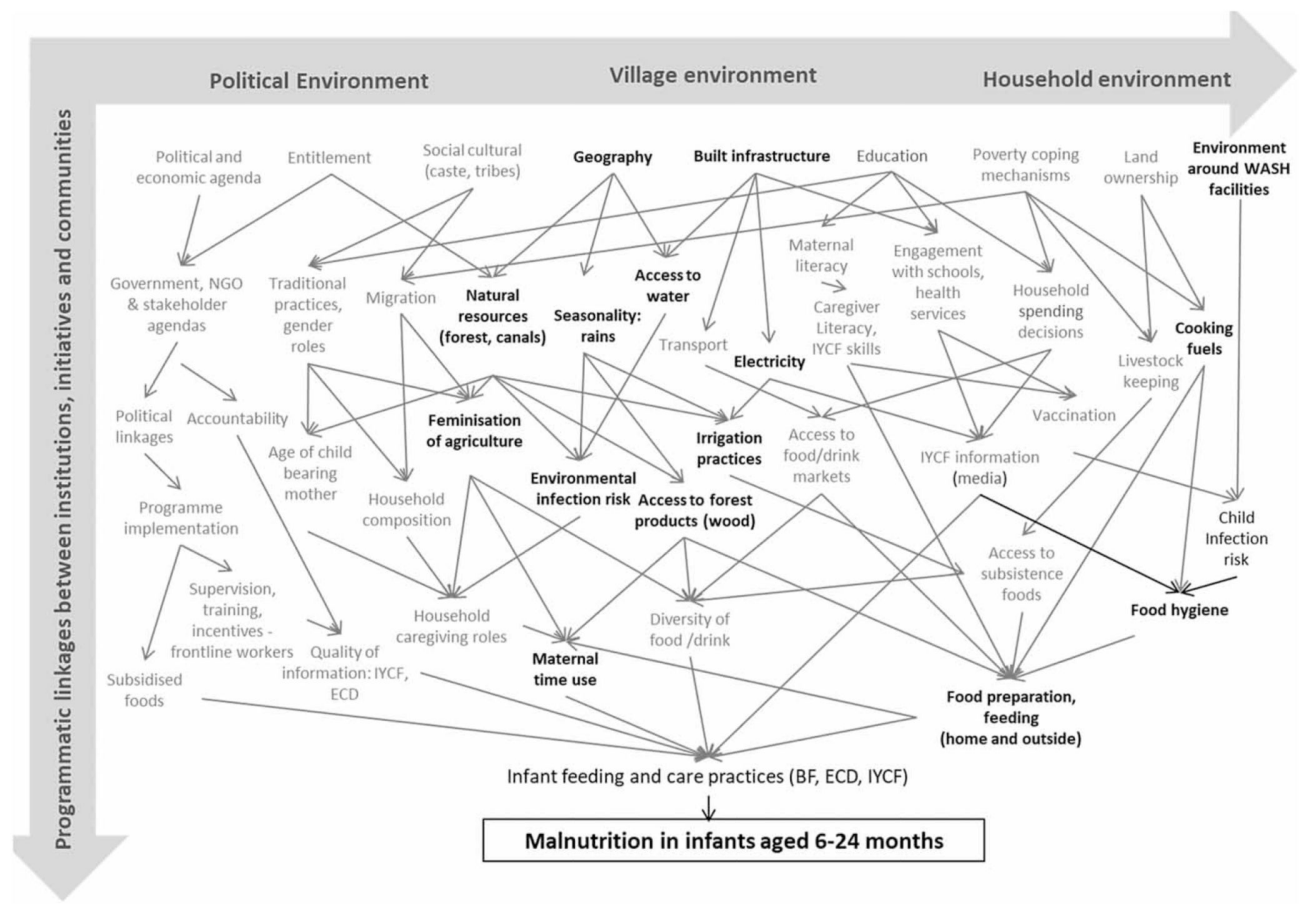
Conceptual framework (adapted from GCRF 2019).

The authors developed a conceptual framework for exploring linkages between feeding practices and interdisciplinary HEEE factors based on the socio-ecological model to explore linkages at multiple levels to consider the political, village/community and household environment (CDC 2019). Figure 2 shows the different factors and the programmatic linkages between institutions, initiatives and communities. The factors in bold font highlight environmental factors at each level. For example, at the village level, resources, infrastructure and time used at the community scale are linked to both the WASH environment and risk of childhood infections. The conceptual framework informed the design of the data collection tools.

### Tools and sampling

Mixed methods were used to evaluate both the level of access of services through household interviews and the potential links to feeding practices through qualitative transect walks. A cross-section of 445 households, with children aged 0–23 months, were interviewed from nine programme villages of Ghatol and Kushalgarh blocks, Banswara district, Rajasthan. See Supplementary information for informed consent forms and household interview templates. Heads of these households provided information about the contextual factors of the house in which they currently live including, but not limited to literacy, occupation, migration for work, water, sanitation, energy, hygiene and livestock.

Between January and April 2018, transect walks were conducted by the core research team and community field researchers in nine villages in Ghatol and Kushalgarh blocks of Rajasthan, India. Field researchers were selected based on demonstrated understanding of the local community and/or nearby areas and their ability to communicate in the local language. During the transect walk, the team documented key physical features, environmental resources and gaps in environmental resourcing using a combination of observation and photographic evidence. A semi-structured broad narrative discussion guide was used as a prompt discussion of the broader implications of environmental resources and potential impact on feeding practices. See Supplementary information for templates used for transect walks and a short summary of the approach. Typically, in each village two to three such discussions took place with four to six residents based on their willingness to participate. Even though the team tried to engage diverse groups, it was predominantly men who participated in the discussion as those public spaces are traditionally dominated by men. The team discussed and noted environmental features (e.g. canals, wells, drains, toilets, water supply, electricity poles, crops, land use type, construction activity if any,location of key buildings, soil characteristics, energy sources for cooking, flooding, road conditions and solid waste management) and challenges associated with environmental resources and services.

### Data analysis

The household interview data were analysed using SPSS and transect walk, and broad narrative results were analysed using thematic analysis under the key headings of WASH and energy services. For testing the significance of difference between categorical variables, we used the Pearson chi-squared test, and for testing the significance of difference between continuous variables, we used the *F*-test.

## Results

This section presents evidence on potential links between environmental factors (WASH and energy) from observational transect walks and quantitative data from household interviews. Quantitative evidence includes socio-demographic profile and access to WASH and energy services.

### Socio-demographic profile

Tables 1 and 2 provide an overview of the socio-demographic factors for 445 households to provide an improved understanding of the communities and settings for this study. Both blocks had a slightly higher proportion of females, which is in part due to the migration of male workers to urban centres in surrounding regions (Table 1). There were no significant differences in the age distribution of total population by block. There is a significant difference in age distribution by gender in the Kushalgarh block.

Table 2 includes literacy and occupational patterns from both blocks. Literacy levels among both males and females were slightly higher in Kushalgarh as compared to the Ghatol block. A larger percentage of the population overall went to school in the Kushalgarh block. Though overall there were no significant variations in literacy levels of population of both the blocks noted. However, the occupational distribution of households is significantly different between the blocks. Whilst agriculture is the main occupation in both blocks, the proportion of unskilled agriculture labourers was higher in the Kushalgarh block. Approximately 7% of the heads of the households in Ghatol were agricultural labourers without any landholding. In the Kushalgarh block, 11% of the heads of households are agricultural labourers without any landholding. Due to the migration of adult men from the house, mothers were also engaged in agricultural activities reducing the time that mothers had available for cooking and feeding children.

### Water,sanitation and hygiene

#### Water access

During transect walks, it was noted that the Ghatol block was located in proximity to the Mahi canal. Overall, the impression was that there was adequate access to water in terms of quantity of water for both drinking and irrigation. The canal water was used for irrigation throughout the year with no tube wells observed during the transect walk. In contrast, water shortages were noted in the Kushalgarh block during summer. During transect walks, the project team saw one poorly maintained pond which catered to the water needs of the community. Irrigation occurred via the pond, which is dependent upon rainwater. Two villages within the Kushalgarh block were located near the canal but only had access to canal water during the rainy season and winter. Water shortages often limit the ability of families to maintain good hygiene practices, wash hands frequently, maintain a clean environment and also impact food preparation leading to poor feeding practices especially as women were primarily responsible for water collection.

Transect walks also revealed that in both blocks, low-lying areas were flooded during the rainy season acting as potential breeding areas for mosquitoes but houses generally were not flooded. The roads in Ghatol were well constructed which reduced the severity of impacts due to flooding in the rainy season. This contrasts with Kushalgarh where the roads flood and limit accessibility during the rainy season. In both blocks, residents perceived a higher rate of illnesses during the rainy season especially during the months of August and September. In almost all the households of both the blocks, adult women were responsible for fetching water for bathing and cleaning utensils.

The level of water services has been quantified through household interviews (Table 3). There were significant differences in both the source of drinking water and availability of water in own dwelling unit between the blocks. The main source of drinking water was from a hand pump delivered to a shared plot or yard for both blocks with 56% coverage in the Ghatol block and 45% in the Kushalgarh block. Only 1.8% of households had access to water sources in own dwelling in the Kushalgarh block compared to 35% in Ghatol.

Respondents from the Ghatol block highlighted that access to water for drinking and irrigation purposes was not equal for all, although 99% of respondents from both the blocks reported that water is available from their main source throughout the year.

There was limited evidence on handling and storage of water though most residents used buckets and pots. Inadequate storage may result in contamination of stored water by users but is unlikely in itself to cause bacterial growth (Waller *et al*. 2020). There was a significant gap in water filtering practices between the two blocks (59% in Ghatol vs 2% in Kushalgarh), with the majority of households who filter water using cloth filters to treat water. Cloth filtering can remove bacteria from water and provide protection against cholera (Huq *et al*. 2010). No data were available on water quality for communal water sources in the villages.

#### Hygiene and sanitation

The level of access to sanitation services was quantified through household interviews (Table 4). Both the blocks differ significantly in terms of access and use of toilets in households. Approximately 37% of households in Ghatol and 58% of households in Kushalgarh blocks did not have household toilets. Only 36% of households in Ghatol and 6% of households in Kushalgarh used toilets for the intended purpose. Nearly 83% of residents in Kushalgarh and 47% in Ghatol reported practising open defecation.

Discussions during the transect walk highlighted barriers around the use of toilets in households. This ranged from economic (costs to build and decorate toilets), social (sharing of toilets with in-laws) and behavioural (used to open defecation). It was observed that residents often built the structure for toilets but then used the structures to store grains or wood. In one instance, pits had been excavated for toilets but the pits were now waterlogged becoming a breeding place for mosquitoes. This had the potential of adversely affecting the health of infants if malaria was contracted through contact with the mother and/or if contracted during play. Ill health through malaria can then lead to further malnutrition making it difficult to break the vicious cycle of malnutrition.

Households did not directly discuss hygiene during the transect walk, but observations suggest most respondents use ash only or ash and soil to wash their hands. The use of soap was rare except for bathing/washing in some instances. Handwashing is dependent on access to water, which was a challenge during summer months. Open defecation was practised in nearby fields with residents then using soil and ash for cleaning hands.

Other forms of contamination may occur if water surfaces are not protected from contamination. This was critical in Ghatol where the shallow water table poses the risk of water contamination. There was also a gap in understanding the characteristics of a safe toilet. For example, a transect walk respondent felt that having a pit latrine is safe but as the pit was not lined, there was a risk of contamination of groundwater leading to the use of contaminated water sources for the preparation of food.

In addition to sanitation for carers, sanitation at all levels is important for creating environments for safe feeding practices. Many studies target households and omit schools and other village-level facilities. Most of the toilets visited in schools were in poor condition with broken doors, in waterlogged fields and were dysfunctional. There was no discussion on managing faeces for infants and safe disposal, though this could be attributed to discomfort discussing this topic indicating a barrier related to awareness and/or openness to discuss this issue.

#### Energy

Energy access for cooking and lighting was assessed through household interviews. There were significant variations between the blocks in terms of fuels used for cooking food. In spite of variations, in both the blocks, the main cooking fuel source was wood (65% in Ghatol and 60% in Kushalgarh) followed by dung cakes (Table 5). In spite of the Government of India Ujjawala scheme to provide subsidised LPG to households, the uptake was slow as noted in Table 5. With no significant variation, only 13–14% of households have the cooking area in a separate room, and more than two-thirds of the respondents experience power cuts (Table 5). Only 37% of households has access to electricity as a source of lighting as noted in household interviews. Seventy percent of houses in both the blocks used kerosene lamps for lighting. Variations in power cut were significantly different, with all the households of Ghatol experienced power cuts when this figure is 78% in Kushalgarh (Table 5).

During transect walks and discussions, one of the barriers to uptake of LPG noted was the high deposit costs required to acquire the canisters in the first instance. Other factors discussed included taste of food and preference for using traditional cooking methods such as with wood and other biofuels. This is important to consider, as more time is required by mothers and other carers to obtain these fuels.

In practice, the electricity supply was erratic and only covered short periods. During transect walks, it was noted that in Ghatol, households have access to electricity for 10 h, whilst in Kushalgarh, they only have access for 2 h/day. During transect walks, the team observed that some houses in Ghatol had television sets dependent on their caste and socio-economic conditions.

## Discussion

### Environmental factors

As IYCF is not just about the quantity and quality of food, it is vital to explore broader environmental factors influencing feeding practices. For example, if the water children drink is unclean, it will be harmful or if the interaction between water and food preparation is unclean (because of inadequate handwashing, where food is prepared, how the utensils are cleaned), children will be at a higher risk of infections. Access to toilets and hygiene practices related to sanitation is linked to both caregivers’ and children’s overall health as handling food without washing hands, for example, can increase the risk of infection.

Sanitation for carers is important to consider for infant nutrition because feeding practices take into account hygiene connected to food preparation: Factors such as handwashing prior to cooking and feeding children will have strong implications for preventing contamination from faeces and other waste (from adult to child). For caregivers looking after children, open defecation practices could open up multiple pathways to infections. Whilst the intent behind the Clean India Mission is to provide access to toilets and safe sanitation for all, the reality on the ground was a gap between policy interventions and local practices.

Waterlogging outside homes increased the likelihood of breeding mosquitoes, which poses a health risk for malaria, and the transmission of other infectious diseases negating the benefits of good feeding practices. Inequity in water access could adversely affect feeding practices within groups, which are already vulnerable and challenged by lack of access to resources. Unlined pit latrines pose a high risk for water quality due to contamination of groundwater, potentially leading to the use of contaminated water in food ingested by infants. The water table in Ghatol was shallowly implying a higher risk of contamination of groundwater potentially affecting food preparation. The links above demonstrate the multiple infection pathways arising out of poor WASH services.

### Socio-cultural factors

Social-cultural factors emerge and raise dynamics such as the role of multi-family and multi-generational caregivers in delivering feeding practices. Agriculture was the dominant profession for heads of households in both blocks which may have implications for the kinds of activities caregivers are involved with and how that can influence feeding practices in the physical environment. In both blocks, mothers would work in fields, more so in the less affluent Kushalgarh block. Because the dominant profession of caregivers (mainly mothers) is engaged in agriculture, in the caregiver’s absence, grandparents often would look after children and be responsible for feeding them. This indicates a context where any nutritional, behavioural and environmental interventions would need to target other family members in addition to mothers.

Collecting wood is a time burden on women and careers, which limits the time available for food preparation and childcare. This can influence methods of cooking food for infants, time taken to cook food and potential health impacts in terms of air pollution.

Very few households had adequate sanitation in the form of safe household toilets placing a burden on caregivers. In some households where toilets are shared between households, elders continued to go to the field for open defecation. This introduces a series of multiple infection pathways: adults without access to clean sanitation combined with a lack of handwashing practices are likely to contaminate children when feeding and caring for them. Caregivers who play a vital role in the life of the child are likely to be exposed themselves and expose infants to multiple pathways for infections when in contact with food, cooking and when feeding children. This kind of intergenerational and social linkage demonstrates why schools, community and family practices have been considered as environments where feeding practices are influenced by a range of factors, experienced differently by caregivers. The implications of taking this diversity of linkages into account are that it can inform efforts to tackle SDG 6 (WASH) as well as SDG 2 (Hunger) and SDG 3 (Health).

### Institutional factors: from policy to delivery

There was a mismatch between the field observations from the transect walks and the ambitions set out by the POSHAN mission. Initiatives such the ‘Clean India Mission’ fund the construction of toilets but the team observed that most households used the toilets as storage rooms or had not completed toilet construction due to both a combination of social, behavioural, institutional and economic barriers. The coverage of the mission was uneven, as households were not clear on how to access resources or government funding was deemed to be inadequate for constructing toilets. On the delivery end, the households who did access the resources did not feel that toilets were appealing in terms of ventilation and physical appearance leading to reduced uptake of the scheme. The fact that schools do not have adequate sanitation facilities poses a health risk for children further highlighting the gap between policy and implementation (GCRF 2019). There are further implications for SDG 5 for achieving gender equality by 2030 as if there are no clean toilets in homes and schools, girls will adversely be affected. Similarly, there were barriers to uptake of clean cooking fuel leading to high consumption of wood and hence a time burden placed on caregivers for the collection of wood.

## Conclusions

The use of observational transect walks enabled capturing the linkages between feeding practices of the households, environmental factors across generations and could account for behaviours in society more broadly. The transect walk was successful in highlighting potential pathways to infection and identifying multiple linkages between inadequate infrastructural services and poor feeding practices.

There were challenges encountered during the transect walks that should be considered for conducting future studies. First, there were some locations where a specific local dialect, Wagdi, was used. The team did have one researcher fluent in this local dialect and all team members were fluent in the local language but this still posed difficulties in the translation and transcription of data. The broad narrative discussions during transact walks were intended to elicit conversations and interactions about environmental conditions. Even though the team tried to engage with both male and female participants, the participants of the discussions during transect walks were male as they predominantly dominate discussions in public spaces. Despite efforts to have a female field researcher, one of the challenges of this study was the ability to involve women in discussions during the transect walk, and further work is required on study designs which are inclusive.

In exploring ways to provide an enabling environment to caregivers for optimal infant feeding practices, the findings suggest interventions such as capacity-building activities to promote the construction of safe sanitation solutions such as lined pits as a pre-requisite to decentralised sanitation systems to mitigate the risk of groundwater contamination. This would need support in the form of training on faecal sludge management and appropriate mechanisms in place to ensure safe disposal and treatment systems for faecal sludge. In the long term, there is a need to explore networked infrastructural solutions with localised wastewater treatment options. Developing appropriate, affordable and acceptable sanitation requires the co-development of solutions for the entire value chain jointly with key stakeholders such as the Clean India Mission officials and community members. Such initiatives should include greater attention to baby wash and clean environment and play areas.

There was a general gap in information in the villages on water quality, and hence there were limited community and household-level interventions to purify or filter water. Setting up a testing regime for water quality by the local authorities and sharing of results on the results regularly would promote investment into water filters and improve practices at the household level. The filling of low-lying spots could reduce flooding and water storage to reduce the risk of malaria.

The findings suggest further work on understanding the social–cultural barriers to uptake of environmental interventions such as uptake of toilets in houses, water scarcity and subsequent impact on daily routines and water use in houses, use of LPG for clean cooking, handwashing with soap and water filtration practices. These studies would enable stakeholders to better develop interventions targeting behaviour change from a contextualised evidence base. This would influence feeding practices within households.

This study demonstrates the potential links between environmental and feeding practices for the setting of Banswara district in Rajasthan. Our study also highlights a gap in policy, aspirations and actual implementation on the ground. Policymakers need to work closely with researchers and local community-based organisations to ensure uptake of policies, assess potential barriers and facilitate opportunities to ensure wider outreach. The POSHAN mission provides an opportunity to integrate interventions targeting nutrition and environment to address the broader contextual socio-cultural barriers to improving feeding practices. Given the strong role of other family members such as grandparents, other caregivers such as siblings, future interventions will need to be holistic and target the household in its entirety in addition to ongoing targeted healthcare interventions for mothers.

Moving forward with approaches like this, one can enable interdisciplinary teams to co-develop harmonised integrated interventions tailored to the needs of local communities. Investing in SDG 6 and SDG 7 would not only improve environmental conditions but also influence IYCF and hence well-being for children (SDG 2, SDG 3 and SDG 5). Environmental interventions therefore can leverage and lead to wide-ranging benefits especially in tandem with targeted public health measures. This would support the UN 2030 Agenda by achieving well-being for all with children at the heart of future interventions.

## Figures and Tables

**Table 1:** Age distribution for Ghatol and Kushalgarh blocks (%)

	Ghatol		Kushalgarh		Total population	
Age	Male	Female	Male	Female	Ghatol	Kushalgarh
0–1	21.7%	15.2%	16.9%	15.4%	18.3%	16.1%
2–4	9.2%	10%	9.7%	10.4%	9.6%	10.1%
5–18	18.3%	20.8%	19.9%	28.4%	19.6%	20.2%
19–29	25.4%	32.5%	27.6%	25.4%	29.0%	26.4%
30–49	18.1%	14.3%	16.9%	13.6%	16.2%	15.1%
≥50	7.4%	7.2%	9.1%	6.9%	7.3%	7.9%
Total	568	600	670	780	1,168	1,450
Mean age (SD)	19.5 (17.1)	19.9 (17.1)	21.1 (17.7)	18.7 (16.5)	19.7 (17.1)	19.6 (17.0)
F-value (*p*-value)	0.16 (*p* < 0.69)		7.1 (*p* < 0.01)		0.10 (*p* < 0.89)	

Source: Household interviews.

**Table 2:** Educational levels and occupation

	Response	Ghatol	Kushalgarh	χ^2^ (*p*-value)
Education head of the household	Illiterate	34.6%	26.8%	6.7 (*p* < 0.08)
	Schooling	59.1%	68.4%	
	Intermediate or diploma	1.4%	0.0%	
	Graduation and above	5.0%	4.8%	
	Total number of HH	217	228	
Occupation head of the household	Unskilled or agricultural labourer	6.9%	11.4%	14.1 (*p* < 0.01)
	Agricultural landowner	90.3%	84.6%	
	Skilled worker in factory or business	0.5%	3.9%	
	Technicians and associate	0.5%	0.0%	
	Professionals	1.8%	0.0%	
	Total number of HH	217	228	–

Source: Household interviews.

**Table 3:** Water access and availability

	Response	Ghatol	Kushalgarh	χ^2^ (*p*-value)
Source of drinking water	Hand pump into dwelling/yard/plot	55.9%	44.7%	55.8 (*p* < 0.000)
	Public hand pump/tube well/borehole	43.2%	32.9%	
	Uncovered dug well	0.5%	22.4%	
	Surface water (river/lake/pond/stream)	0.5%	0.0%	
Water source in dwelling	Yes	35.2%	1.8%	82.0 (*p* < 0.000)
Water availability from source entire year?	Yes	99.0%	99.1%	0.14 (*p* < 0.71)
Who collects water for bathing/cleaning utensils	Adult woman ≥ 15 years	99.5%	99.6%	0.002 (*p* < 0.97)
	Adult man ≥ 15 years	0.5%	0.4%	
Steps taken to make water safe for drinking?	Yes	58.7%	1.8%	168.5 (*p* < 0.000)
Total households		217	228	–

Source: Household interviews.

**Table 4:** Sanitation and hygiene factors

Blocks	Ghatol	Kushalgarh	χ^2^ (*p*-value)
Absence of toilet	37.6%	58.3%	18.0 (*p* < 0.0001)
Presence of toilet/no use	26.3%	35.1%	3.7 (*p* < 0.06)
Toilets in use	36.2%	6.6%	57.6 (*p* < 0.001)
Open defecation	46%	82.5%	62.8 (*p* < 0.001)
Total households	217	228	–

Source: Household interviews.

**Table 5:** Energy sources and availability

Energy consumption	Response	Ghatol	Kushalgarh	χ ^2^ (*p*-value)
Type of cooking fuel used	Electricity	1.0%	0.0%	20.0 (*p* < 0.001)
	LPG/natural gas	6.2%	0.4%	
	Biogas	1.4%	1.3%	
	Wood	65.1%	60.2%	
	Agriculture waste	0.5%	0%	
	Dung cakes	25.8%	38.1%	
Main lighting source	Electricity	37.5%	37.8%	
	Kerosene	58.5%	62.2%	
Separate room used as a kitchen	Yes	13.0%	14.0%	0.12 (*p* < 0.73)
Power cuts	Yes	100%	78.3%	15.7 (*p* < 0.0001)
Total households		217	228	

Source: Household interviews.

## Data Availability

Data cannot be made publicly available; readers should contact the corresponding author for details.

## Highlights

- The use of observational transect walks to better capture linkages between environment and feeding practices.
- The socio-ecological framework is used to map links between built environment and feeding practices at the village, household and individual level.
- Opportunities to improve child health through linkages to SDG 2 (Hunger), SDG 3 (Health), SDG 6 (Baby WASH) and SDG 7 (Energy).
